# Stochastic Resonance in Insulator-Metal-Transition Systems

**DOI:** 10.1038/s41598-020-62537-3

**Published:** 2020-03-26

**Authors:** Bitan Bhar, Abhishek Khanna, Abhinav Parihar, Suman Datta, Arijit Raychowdhury

**Affiliations:** 1 School of Electrical and Computer Engineering, Georgia Institute of Technology, Atlanta, GA 30332 USA; 2 Department of Electrical Engineering, University of Notre Dame, Notre Dame, IN 46556 USA

**Keywords:** Biotechnology, Neuroscience, Engineering, Mathematics and computing

## Abstract

Stochastic resonance (SR) is an ingenious phenomenon observed in nature and in biological systems but has seen very few practical applications in engineering. It has been observed and analyzed in widely different natural phenomenon including in bio-organisms (e.g. Mechanoreceptor of crayfish) and in environmental sciences (e.g. the periodic occurrence of ice ages). The main idea behind SR seems quite unorthodox – it proposes that noise, that is intrinsically present in a system or is extrinsically added, can help enhance the signal power at the output, in a desired frequency range. Despite its promise and ubiquitous presence in nature, SR has not been successively harnessed in engineering applications. In this work, we demonstrate both experimentally as well as theoretically how the intrinsic threshold noise of an insulator-metal-transition (IMT) material can enable SR. We borrow inspiration from natural systems which use SR to detect and amplify low-amplitude signals, to demonstrate how a simple electrical circuit which uses an IMT device can exploit SR in engineering applications. We explore two such applications: one of them utilizes noise to correctly transmit signals corresponding to different vowel sounds akin to auditory nerves, without amplifying the amplitude of the input audio sound. This finds applications in cochlear implants where ultra-low power consumption is a primary requirement. The second application leverages the frequency response of SR, where the loss of resonance at out-of-band frequencies is used. We demonstrate how to provide frequency selectivity by tuning an extrinsically added noise to the system.

## Introduction

Noise is an omnipresent yet unwanted characteristic of all natural systems. Since we cannot eliminate noise from useful signals, the commonly used engineering technique is to have a stochastic estimate of the noise and design engineering solutions that can improve the signal-to-noise ratio and reduce the overall impact of noise. In contrast, the motivation behind stochastic resonance (SR) is to harness the noise power in an intelligent way, similar to certain natural systems, to enable better engineering solutions. In the context of electrical systems, we are mostly interested to amplify a low-amplitude input signal with the help of noise; instead of the noise acting as distortion that reduces the information content of the signal. This enables us to transmit and detect low power signals through a noisy channel. The phenomena of SR has been observed and studied in diverse fields such as biological systems^1,2^, global climatic studies^3–5^ and theoretical physics^6–8^ etc. The main aim of this article is to demonstrate possible engineering applications of this natural phenomena, instances of which are can be found in^9–11^. The phenomenon of SR in a multistable system and its application in fault-diagnosis in mechanical systems has been discussed in^12,13^. Further^14^ and^15^ provide an extensive discussion about the use of SR in enhancement of energy harvesting in electromechanical systems.

**Figure 1:**
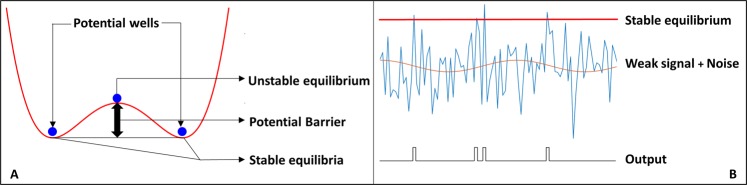
Stochastic Resonance (**A**) Bi-stable System exhibiting Classical Stochastic Resonance (**B**) Excitable system showing Stochastic Resonance.

There are two main kinds of stochastic resonance that have been explored^16^. Classical SR requires three main components, namely a bi-stable potential-well system (Fig. 1A), a weak signal whose power is insufficient to make the transition from one well to another and a noise source which enables the signal to overcome this potential barrier and make spontaneous transitions^17^. One example of such a system is of the form:

1 $$\dot{x}=-\,U{\prime} (x)+Asin(2\pi ft)+\eta \sigma (t)$$

where the bi-stable potential profile, U(x) is described by:

2 $$U(x)=\frac{{x}^{4}}{4}-\frac{{x}^{2}}{2}$$

Here, A is the amplitude of a sinusoidal signal, and σ(t) is the noise process with unit variance. The dynamical system has two stable equilibria namely at x= ±1, and an unstable equilibrium at 0. If the value of A is less that what is required to cross the potential barrier the system will stay at one of the stable states without any external influence. It has been shown^6^ that if the noise variance is in a certain range (*η*_1_, *η*_2_), the output oscillates between −1 and +1 at the frequency of the signal. The lower limit of the noise variance comes from the fact that at lower values of the noise power, the system does not have enough energy to overcome the potential barrier and make spontaneous transitions. Further, if the variance is too high the system will cross the barrier irrespective of the signal value (amplitude) and the output will have no correlation with the input, thereby breaking the notion of resonance. The theoretical aspects of SR in bi-stable systems has been studied in^6,7,17,18^. The Fitz-Hugh-Nagumo Model is capable of acting as a bi-stable, excitable or oscillatory system for different parameters, and therefore provides a great way to exhibit Stochastic Resonance.

The second type of stochastic resonance does not require a bi-stable system. Instead it requires an excitable system with a thresholding mechanism, where if the input crosses a certain value we record one event such as a spike at the output (Fig. 1B). In an SR driven excitable system, the input signal alone cannot provide a spike, but in conjunction with noise the system gains enough excitatory power to produce a temporary output and eventually relaxes back to the equilibrium state^19,20^. The relaxation process is governed by the system parameters. So we see a spike at the output whenever the noise crosses the threshold. Much like the previous case, the noise power should be bounded to avoid being stuck in the stable state and also prevent spontaneous spike generation uncorrelated with the input. In this paper, we demonstrate SR of the second kind where an electronic system is designed to show SR driven excitatory behavior, capable of performing signal-processing.

The term SR has also been used by authors in linear systems where the output of the system varies as a function of some particular characteristics of the noise^21^. shows and calculates SR in case of linear sustems with multiplicative noise where the SNR of the system exhibit extrema as the noise correlation time and asymmetry changes.

## System Modeling

**Figure 2:**
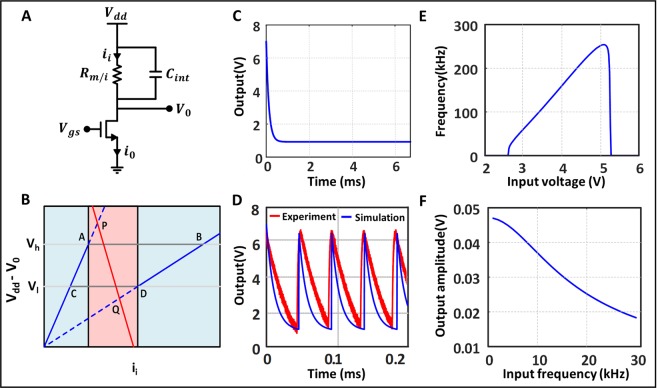
Illustration of the characteristics of the circuit model. (**A**) The equivalent circuit representation of our model. *V*_*gs*_ denotes the input node and *V*_0_ the output. The IMT device shows two resistances namely *r*_*m*_ and *r*_*i*_ depending on its state. (**B**) The null cline for the model. CAB and BDC denote the transition from insulating to metallic and metallic to insulator states respectively. PQ denotes the load-line for the CMOS in saturation. The two indigo regions denote the stable states and the pink region denotes the oscillatory state. The actual state of the system is determined by where the CMOS load-line crosses the device lines. (**C**,**D**) show the voltage output at the stable and oscillatory states respectively while the red and blue lines in the later show experimental and simulated values respectively. (**E**) The natural frequency of oscillation of the system in the oscillatory state as a function of a constant input voltage. It shows that the system is oscillatory only for a certain range of input voltage. (**F**) shows output amplitude vs frequency when the input is biased at a voltage inside the insulating region (left indigo region in **B**) and a sinusoid of amplitude 0.01 V added to that. It shows that the gain of the system goes down with frequency.

The circuit primitive (Fig. 2A) that produces SR is composed of an IMT device composed of vanadium-dioxide (VO_2_)^22^. The VO_2_ device has a pull-down metal-oxide-semiconductor (MOS) transistor that produces load-line characteristics. The VO_2_ device has two electrical states, namely a metallic (M) state and an insulating (I) state. It acts as a resistor in both these states, but the resistance in the insulating state (line AC in Fig. 2B) is much higher than that of its metallic state (line BD). The device spontaneously switches from one state to another depending on the voltage applied across it. When the voltage across the device reaches an upper threshold, *ν*_*h*_ (point A), the device transitions from insulating to metallic state. Correspondingly when the voltage across the device falls to a lower threshold, *ν*_*l*_, it goes back to the insulating state (point D). Further, the device shows hysteresis, *ν*_*h*_ > *ν*_*l*_ (closed loop CABD, Fig. 2B). The capacitance C_int_ captures the cumulative effect of all the capacitive effects present in the circuit.

The state equations for the circuit can be written as:

3 $${v}_{0}=({v}_{dd}-h({i}_{i},s))$$

4 $${C}_{int}\frac{d({v}_{dd}-{v}_{0})}{dt}={i}_{0}-{i}_{i}$$

where ${v}_{0}$ and ${v}_{dd}$ denote the output and supply voltage respectively as shown in Fig. (2A), s denote the state of the IMT device, i.e. $h({i}_{i},s)={R}_{m}{i}_{i}$, when it is in the metallic state and $h({i}_{i},s)={R}_{i}{i}_{i}$ when it is in its insulating state (${R}_{m}$ and ${R}_{i}$ denoting the resistance offered by the device in its metallic and insulating state respectively with ${R}_{m}$≪..), ${i}_{i}$ is the current through IMT device, and ${i}_{0}$ is the current through the MOSFET. The null-clines of these equations along with the circuit model is shown in the Fig. (2B).

The orange line in (B) represents the load line for the circuit given by the second equation. If this line crosses the null cline for the device somewhere to the right of D (metallic state) or to the left of A (insulating state), the system has one stable state (Fig. (2C)). Once the system reaches that state, it remains there (Fig. (2C)). But if the load-line crosses the device V-I curves in between A and D, the system has no stable states and continues to follow the trajectory ABDC in the state space (shown in Fig. (2B)). The system is therefore in an oscillatory state, and continuously oscillates between the metallic and insulating states (Fig. (2D)). Intuitively, when the voltage across the VO_2_ device becomes more than ${v}_{h}$, it goes into its metallic state. Consequently, its resistance becomes low and the internal capacitance get discharged thereby pulling the output voltage towards ${V}_{dd}$. But as ${v}_{0}$ approaches ${V}_{dd}$ the voltage across the device becomes too low and when it is less than ${v}_{l}$ the resistance of the device is switches to an insulating and the discharging process stops. The capacitor is now being charged by the current through the MOS and the whole cycle repeats.

The frequency of oscillation can be estimated by assuming that the inductive effects and the current through the device in the insulating state have negligible effect on the dynamics. Therefore setting ${i}_{i}$ to 0 and assuming that the MOSFET is in saturation while charging the capacitor Eq. (4) simplifies to:

5 $${C}_{int}\frac{d({v}_{dd}-{v}_{0})}{dt}={i}_{0}=\frac{\mu }{2}{{v}_{in}}^{2}(1+\lambda {v}_{0})$$

${i}_{0}$ in this case is the saturation current as the MOS is now in saturation. $\mu ={\mu }_{n}{C}_{ox}\frac{W}{L}$ denote the combined NMOS parameter, *λ* denotes the channel-length modulation parameter and the input voltage is ${v}_{in}={v}_{gs}-{v}_{th}$.

This can be solved to find the approximate fall time of the output as:

6 $${T}_{f}=\frac{2{C}_{int}}{\mu {{v}_{in}}^{2}\lambda }\,\mathrm{ln}\,\frac{{v}_{dd}-{v}_{l}+\frac{1}{\lambda }}{{v}_{dd}-{v}_{h}+\frac{1}{\lambda }}$$

The capacitor discharges through the metallic path when the device is in metallic state. As the voltage across the transistor is very low, ${i}_{0}$ is negligible. Hence,

7 $${C}_{int}\frac{d({v}_{dd}-{v}_{0})}{dt}=-\,\frac{{v}_{dd}-{v}_{0}}{{R}_{m}}$$

Therefore, the rise time is given by, ${T}_{r}={R}_{m}{C}_{int}\,\mathrm{ln}\,\frac{{v}_{h}}{{v}_{l}}$. The fundamental frequency of oscillation for a constant ${v}_{gs}$ is therefore

8 $${f}_{F}=\frac{1}{{T}_{f}+{T}_{r}}$$

The dependence of ${f}_{F}$ with ${v}_{in}$ is shown in Fig. (2E). As we increase the input voltage the MOS current increases, therefore the capacitor charges faster and the frequency of oscillation increases, until the voltage is so high that ${v}_{0}$ itself saturates at a voltage close to ${V}_{dd}$ and the oscillations stop. Our system shows similar behavior to the linearized Fitz-Hugh-Nagumo model of a neuron as has been shown in^18^. Also the current through the device is therefore almost negligible when the device is in the insulating state (experimentally measured at ~50 $\mu A$) and when the state of the device switches to metallic we measured a large switching current which can be approximated by $\frac{{v}_{dd}-{v}_{0}}{{R}_{m}}$. Experimentally, we observe this current to be about 6 mA and it discharges the capacitor quickly and collapses to a low value as the IMT device enters the insulating state.

To understand SR in the current electrical system, let us consider a periodic sinusoidal input signal to the system as given by:

9 $${v}_{in}={V}_{dc}+Acos(2\pi {f}_{in}t)$$

We consider two main sources of noise in this system. The first one is intrinsic and can be described as the random ${v}_{h}$ fluctuation arising from thermal and shot noise sources. The second noise source is extrinsic and is present at the input ${v}_{in}$. We explore the effect of both these noises in the following segments. It will be seen that both of them have similar qualitative effect on the system, but differ quantitatively as they undergo different noise-transfer-functions (NTFs). In the following discussion we have assumed that the noise has a Gaussian profile. This allows us to develop experimentally verified theoretical models. But at the same time it should be noted that other noise models can also be used in the treatment of SR, either numerically or analytically.

**Figure 3:**
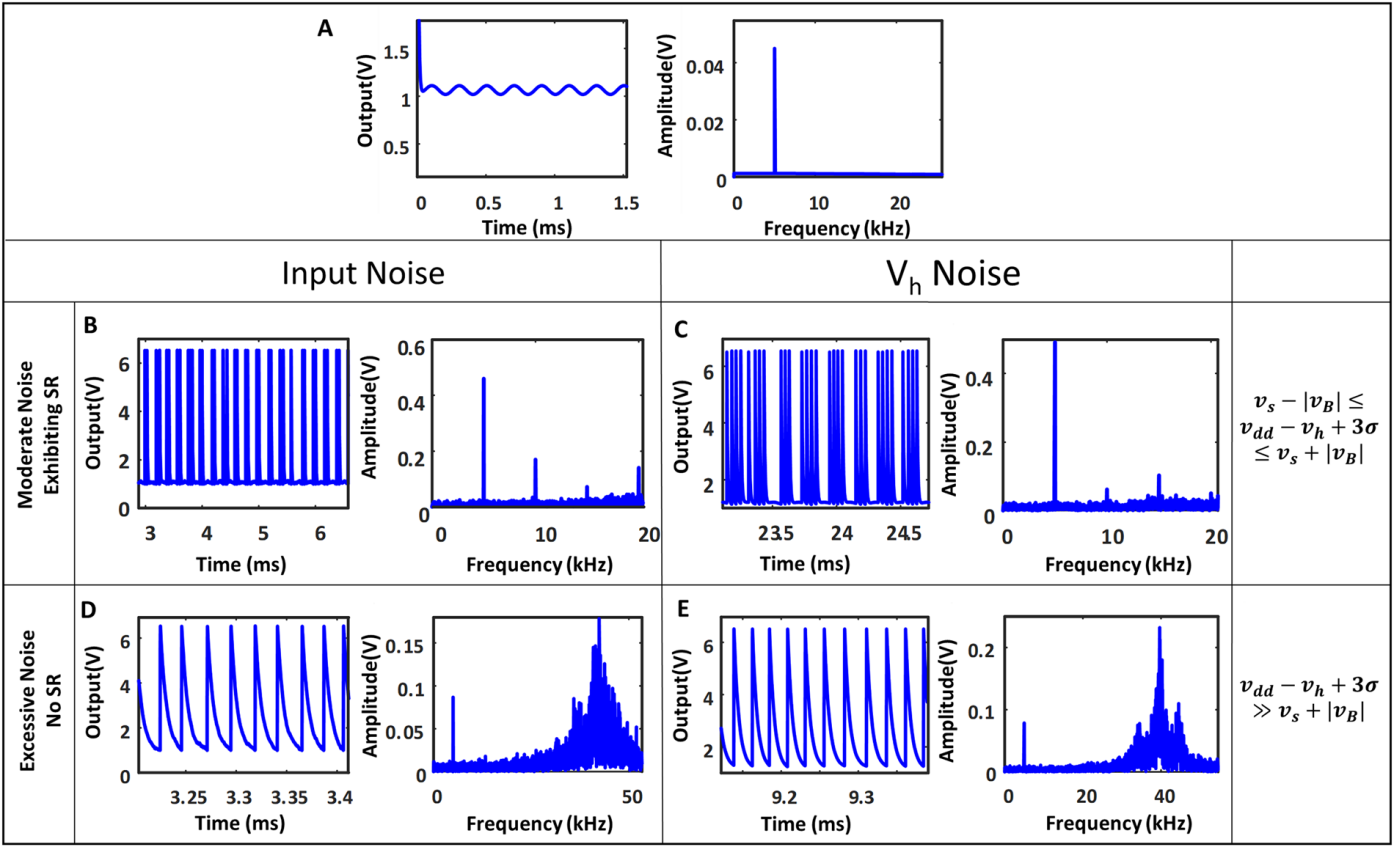
Effect of noise at the input and at the upper threshold of the device in time and frequency domain respectively. (**A**) The time and frequency domain output of the system for input ${v}_{in}={v}_{gs}+Acos(2\pi ft)$, where ${v}_{in}=2.6$ volts and A = 0.01, such that the system does not go in the oscillatory zone. (**B**) Shows the SR in effect with noise added to the input with standard deviation 0.13. (**D**) Shows the effect of adding excessive noise (in this case 0.4). (**C**) Shows the same as (**B**) wit_h_ the noise source now being V_h_, where ${v}_{in}$=2.58 volts and noise standard deviation 0.05, where as (**E**) shows the effect of excessive V_h_ noise with noise standard deviation 0.1.

First let us examine the scenario where there is negligible noise. The value of ${V}_{dc}$ in (9) is chosen in such a way so that the output of the system for the sinusoidal input is unable to cross the threshold for spontaneous oscillations. The circuit acts as a trans-conductance amplifier with a frequency-dependent gain. In the current design, the gain of the system is small and hence the output signal has the same order of magnitude as the input as shown in the Fig. (3A). The simulated output amplitude for different frequencies with the input amplitude being 0.01 V is shown in the Fig. (2F). We can see that the system exhibits a low-pass behavior.

Now let’s consider the case where the system is perturbed by noise. ${v}_{gs}$ is fixed such that without noise ${v}_{dd}$ − ${v}_{0}$ is less than but close to ${v}_{h}$, so the output is a sinusoid of small amplitude but there is no oscillations yet. We start to increase the noise power. As we increase the noise level value of ${v}_{0}$ starts to fluctuate, the probability to cross the threshold ${v}_{h}$ increases and we can observe spikes as shown (Fig. (3B)). At a moderate noise level we can see that there is a high probability of spiking at the troughs of the input sinusoid and a low probability for the crests; and hence we see a number of spikes in each of the period of the input signal (Fig. 3B,C). As the spikes are centered on the troughs of the sinusoid the principle frequency component of the output is same as the input frequency which is also evident from the frequency spectrum of the output. If the noise is further increased, the probability of spiking increases and loses any correlation with the input (Fig. 3D,E). Consequently, the output amplitude at the input frequency falls off sharply.

## Variation with noise power

**Figure 4:**
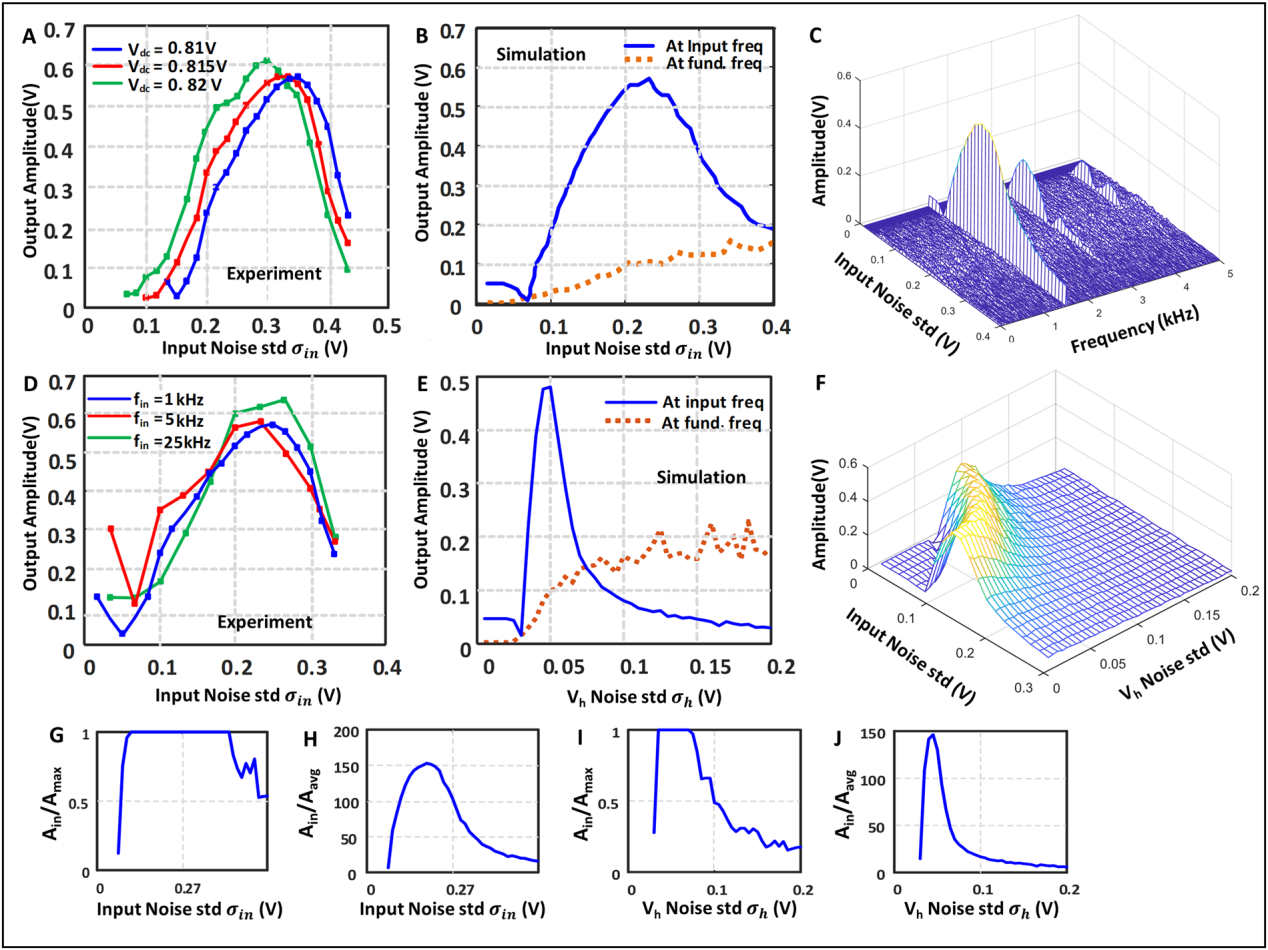
Dependence of the output amplitude on the input and V_h_ noises. (**A**) Shows the experimental results how output amplitude changes with input noise for three bias points. (**B**) Shows the similar thing from simulation, along with how the power at the fundamental frequency varies with the noise. (**G**,**H**) Show the relative strength of the input frequency compared to the maximum amplitude and the average amplitude respectively in presence of the input noise. (**C**) Shows how the whole frequency spectra changes with noise. (**D**) is similar to (**A**) but it shows the experimental data for 3 different input frequencies. (**E**) Shows the simulation results for how output amplitude varies with the V_h_ noise. (**I**,**J**) Show the relative strength of this input frequency compared to the maximum amplitude and the average amplitude respectively in presence of the V_h_ noise. (**F**) Shows the effect of both the noise simultaneously on the output amplitude.

Ideally we want the output power of the system to be at the same frequency as the input. From our discussions, it is clear that low levels of noise will result in low output noise power. At the same time, high noise power will result is lower outout power at the desired freqeuncy. Between these two extremes, the output will exhibit significant power at the input frequency as shown in the Fig. 3B,C. The variation of the output amplitude with the noise power is shown in Fig. 4 with Fig. 4A,D showing experimental results and Fig. 4B,E showing simulation results for the input noise and the ${V}_{h}$ noise respectively. The plot shows the exact characteristics of SR, where the signal is amplified when the noise to the system is in between a certain range. The orange line in (4B) denotes the power at the fundamental frequency of the oscillator. We note that when the noise is too large there are continouos spikes and the power shifts from the input frequency to the fundamental frequency as shown in Eq. (8).

Solving for the fixed points from the two governing Eqs. (3) and (4) of our system the steady state solution to the system for a fixed ${v}_{in}={V}_{dc}$ is given by

10 $${v}_{s}=\frac{{v}_{dd}-\frac{\mu }{2}{{V}_{dc}}^{2}{R}_{i}}{1+\frac{\mu }{2}{{V}_{dc}}^{2}\lambda {R}_{i}}\,{\rm{and}}\,{i}_{s}=\frac{\frac{\mu }{2}{{V}_{dc}}^{2}(1+\lambda {v}_{dd})}{1+\frac{\mu }{2}{{V}_{dc}}^{2}\lambda {R}_{i}}$$

Since our input is ${v}_{in}={V}_{dc}+Acos(2\pi {f}_{in}t)$, if *A* is small the output of the system will be the steady state output for ${V}_{dc}$ plus a sinusoidal component; which can be expressed as ${v}_{O}={v}_{S}+{v}_{B}cos(2\pi {f}_{in}t+\theta )$. As we have an expression for the steady-state output voltage, assuming the input signal amplitude *A* to be inside the small signal range, we can estimate ${v}_{B}$ as the input amplitude amplified by the voltage gain of the system $\frac{d{v}_{s}}{d{V}_{dc}}$. Hence, we can write ${v}_{B}$ as:

11 $$|{v}_{B}|\cong A|\frac{d{v}_{s}}{d{V}_{dc}}|=A|\frac{\left(1+\frac{\mu }{2}{{V}_{dc}}^{2}\lambda {R}_{i}\right)(-\mu {V}_{dc}{R}_{i})-\left({v}_{dd}-\frac{\mu }{2}{{V}_{dc}}^{2}{R}_{i}\right)(\mu {V}_{dc}\lambda {R}_{i})}{{\left(1+\frac{\mu }{2}{{V}_{dc}}^{2}\lambda {R}_{i}\right)}^{2}}|=A\frac{\mu {R}_{i}{V}_{dc}(1+\lambda {v}_{dd})}{{\left(1+\frac{\mu }{2}{{V}_{dc}}^{2}\lambda {R}_{i}\right)}^{2}}$$

For our simulation a typical value of ${v}_{B}$ is ~0.0294 *V* for $A=0.01V$ which matches the experimental results. ${v}_{B}$ also changes with the frequency of the input signal whose value is plotted as a function of frequency in Fig. 2F.

The ideal noise level for harnessing resonance will be such that the system should go in the oscillating mode only when ${v}_{0}$ is near its crest. If the noise level is so low that even the crest of ${v}_{0}$ does not reach ${v}_{dd}-{v}_{h}$ level, there will be no oscillation or resonance. On the other hand, if the noise is so high that the probability of crossing the threshold is almost similar in both the crest and the trough of ${v}_{0}$ then the output will be independent of the input frequency.

Roughly, we can say that the minimum ${v}_{h}$ noise standard deviation ${\sigma }_{min}$ should obey ${v}_{s}-|{v}_{B}|={v}_{dd}-{v}_{h}+3{\sigma }_{min}$, as the chances of noise fluctuations having values more than 3 ${\sigma }_{min}$ is 0.27% and can be safely neglected. For the input noise case all noise standard deviations should be multiplied by the gain of the system like Eq. (11) as it passes through the transistor gain unlike the ${v}_{h}$ noise which just get added to ${v}_{0}$.

And the max value of noise standard deviation ${\sigma }_{max}$ can be roughly given by ${v}_{s}+|{v}_{B}|={v}_{dd}-{v}_{h}+3{\sigma }_{max}$, since higher levels of noise decorrelates the output power from the input signal frequency.

When the system is in the oscillatory region the output is not a sinusoid anymore, so we take the Fourier transform of the signal. Let ${A}_{in}$ be the coefficient of the input frequency component and ${A}_{max}$ be the max coefficient among all frequencies. ${A}_{in}/{A}_{max}$ gives a quantitative idea whether the input frequency is the strongest component in the output, which are plotted in Fig. 4G,I. To harvest max power from stochasticity we should work in a region where ${A}_{in}/{A}_{max}$ is 1. ${A}_{in}$ divided by the average amplitude of all frequencies ${A}_{in}/{A}_{avg}$ denotes how much the input frequency is dominant among all frequencies and can also be used as a useful metric which are shown in (Fig. 4H,J), which should ideally be as high as possible.

The whole frequency spectrum of the output is shown in Fig. 4C when the input noise is varied. We can see that the noise only enhances the input frequency and its harmonics, though at a lesser degree. This shows an important aspect of stochastic resonance as the noise do not enhance all frequencies but a particular one. Figure 4F show the power at input frequency as both the noises are present in the system. When one of the noise is large the amount of the other noise required for Stochastic Resonance decreases, as expected.

## Variation with frequency

**Figure 5:**
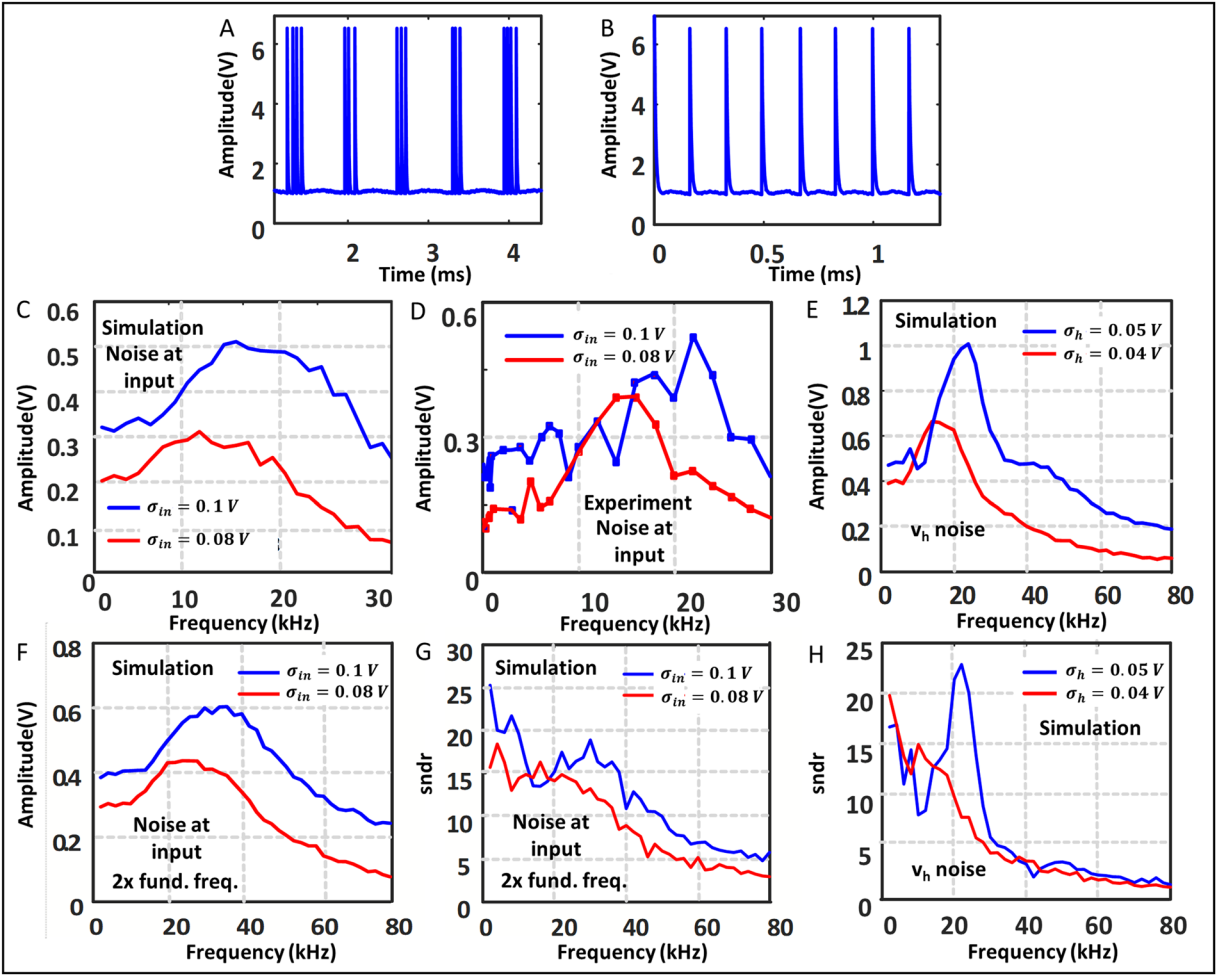
Dependence of output on input frequency. (**A**) Output of the system with moderate (1.5 kHz) input frequency (**B**) Output with large (6 kHz) input frequency. (**C**,**D**) Show how output amplitude changes with input frequency for two different input noise levels from the simulation and the experiment respectively. The fundamental frequency is around 25 kHz for these. (**F**) Shows the amplitude vs frequency with the fundamental frequency double of that of (**C**). (**G**) Show the ratio of the amplitudes of the fundamental frequency and the largest frequency component other than the fundamental frequency corresponding to (**F**). (**E**,**H**) Are similar to (**F**,**G**) but the noise source is now the V_h_ noise instead of the input noise.

The Fig. 5 shows the variation of output amplitude with the input frequency for a fixed noise. The plot shows three major parts: for very low frequencies the output amplitude is quite constant. One such case is shown in the Fig. 5A. We can have an estimate of the number of spikes per period of the input signal as following: assume the first spike starts to appear around the region where ${v}_{s}-{v}_{B}cos(2\pi {f}_{in}t)={v}_{dd}-{v}_{h}+3{\sigma }_{w}$ and after that boundary there are continuous spikes when ${v}_{s}-{v}_{B}cos(2\pi {f}_{in}t)\le {v}_{dd}-{v}_{h}+3{\sigma }_{w}$(where ${\sigma }_{w}$ is the input noise standard deviation ${\sigma }_{in}$ multiplied by the gain of the system in case of input noise or just the ${v}_{h}$ noise standard deviation ${\sigma }_{h}$ in case of ${v}_{h}$ noise) This last part is not strictly true, but taking into account the fact that ${v}_{s}-{v}_{B}cos(2\pi {f}_{in}t)$ is even lower than its previous value the wait time between spikes is negligible and the approximation is quite reasonable. Therefore, the permissible time for spikes per period is given by

12 $$T=\frac{1}{\pi {f}_{in}}{\cos }^{-1}(\frac{{v}_{s}-{v}_{dd}+{v}_{h}+3{\sigma }_{w}}{{v}_{B}})$$

hence, the approximate number of spikes is given by, $N=\frac{T}{{T}_{sp}}$, where ${T}_{sp}$ is the total charging-discharging time for one spike. At this range of low frequencies, ${v}_{B}$ is almost constant; hence N is inversely proportional to the frequency. If there are N spikes in each perod of the signal then as ${T}_{sp}$ is much smaller compared to the time period of the input signal, we can consider the output as a sum of N different impulse trains each shifted from the previous by an amount ${T}_{sp}$ with the strength of each impulse being $\Delta$ where $\Delta$ is the area under one spike. The frequency response of an impulse train of strength $\Delta$ is another impulse train with strength f $\Delta$, since we have N shifted versions of the same. The shift in time by ${T}_{sp}$ is equivalent to multiplication by ${e}^{-j2\pi f{T}_{sp}}$ in the frequency domain; and the output amplitude at the input frequency can be written as:

$${f}_{in}\Delta (1+{e}^{-j2\pi f{T}_{sp}}+{e}^{-j2\pi f2{T}_{sp}}+\ldots +{e}^{-j2\pi f(N-1){T}_{sp}})={f}_{in}\Delta .\frac{1-{e}^{-j2\pi fN{T}_{sp}}}{1-{e}^{-j2\pi f{T}_{sp}}}={f}_{in}\Delta .\frac{{e}^{-j\pi fN{T}_{sp}}}{{e}^{-j\pi f{T}_{sp}}}.\frac{\sin (\pi fN{T}_{sp})}{\sin (\pi f{T}_{sp})}$$

The output amplitude at that frquency is therefore given by $K={f}_{in}\Delta \frac{\sin (\pi fN{T}_{sp})}{\pi f{T}_{sp}}$, which can be approximated to $K\approx {f}_{in}\Delta N=\frac{\Delta }{\pi {T}_{sp}}{\cos }^{-1}(\frac{{v}_{s}-{v}_{dd}+{v}_{h}+3\sigma }{{v}_{B}})$, and is independent of f.

The second part is important when the frequency is such that T is of the order of ${T}_{sp}$ and there is only one spike per cycle. In this case, the permissible time for spikes is so small that if there is one spike in a particular period, then there cannot be any more spikes in the same period when the system relaxes back to the steady state. Hence the output is given by $v={f}_{in}\Delta$ as there is just one equivalent train of spikes, and we can see that output grows linearly with frequency.

And last, when the frequency is comparable to the oscillator frequency, there are not even one spike per period due to the facts that (1) T is now very low hence the probability for spiking is also very low and (2) due to the low pass nature of our circuit, which means at high freuency the output is greatly attenuated and the same noise is unable to cause it to cross the threshold for spiking. Similar effects are seen when we insert noise at the input instead of at the ${v}_{h}$ level. The effect is exactly similar except from the actual values of the noise power.

Figure 5C,D show that if the input frequency is increased for low noises the output falls very fast as there are no spikes for higher frequencies. But for larger noise the power at the input frequency is still quite large. This large amplitude will be useless however, if there are other significant peaks in the frequency spectrum which are very close to the input frequency or even less than it. Figure 5G shows the ratio of the amplitudes of the input frequency to the largest peak other than the input that is less than twice the input frequency. We see after a certain frequency level this falls off quite fast signifying that there are significant power allotted to frequencies that are close to or even less than input, which means for SR purposes we should never operate in these regions.

## Application of SR in IMT devices

**Figure 6:**
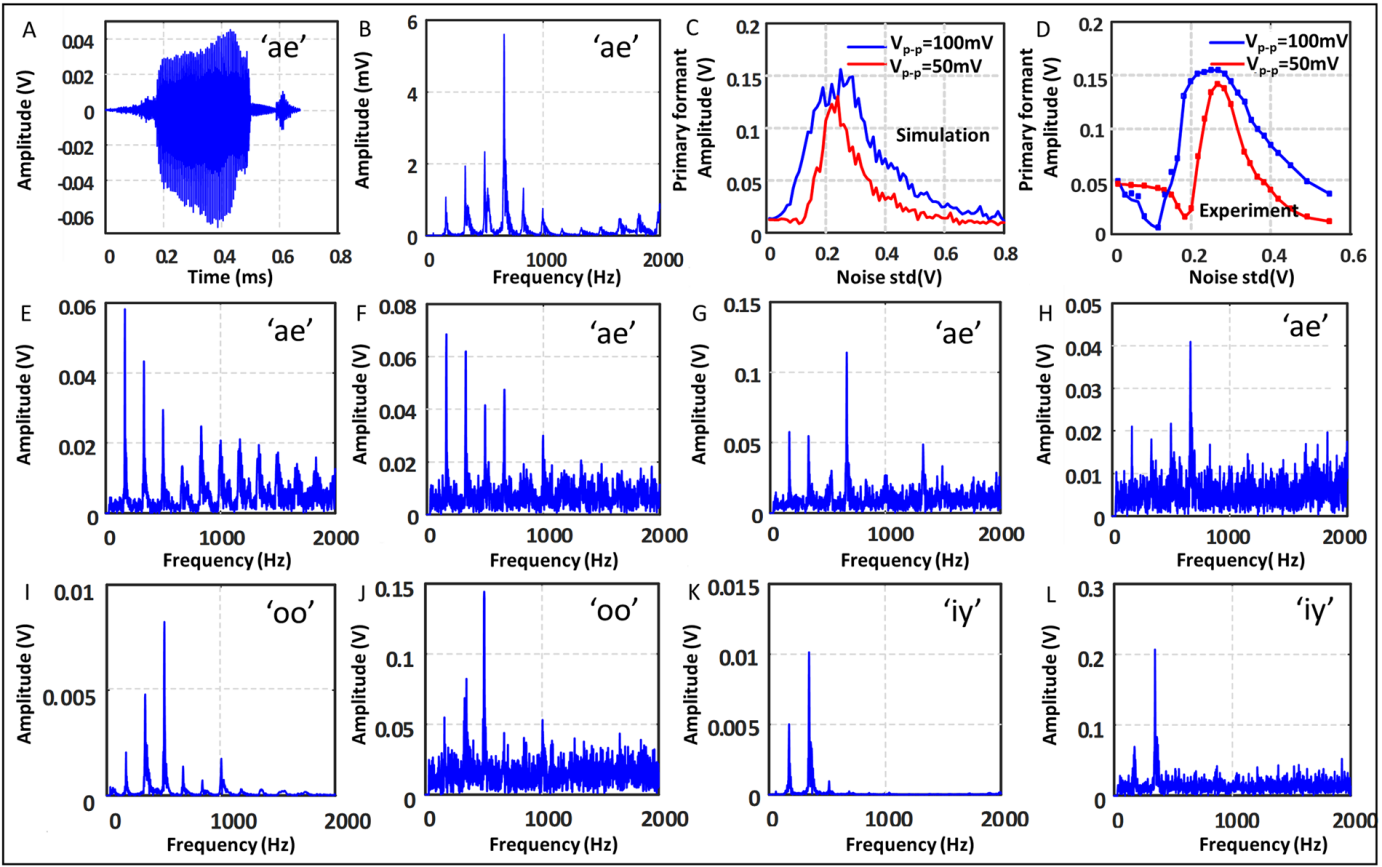
Effect of noise for thresholding vowel sounds. (**A**,**B**) Show the time and frequency domain representation of the vowel sound ‘ae’. (**C**,**D**) Show the power at the primary formant of the vowel at different noise level from the simulations and the experiments respectively for two different amplification of the input signal. (**E**) Show the frequency spectrum of the same vowel after passing through the neuron with amplitude 0.1 V and bias 2.59 V with no noise. (**F**,**G**,**H**) Show the same vowel after thresholding but with noise 0.1, 0.2 and 0.5 V added respectively. (**I**,**K**) Show the frequency spectra for two more vowel sounds namely ‘oo’ and ‘iy’. (**J**,**L**) Show the corresponding outputs from the system with input noise fixed at 0.2.

Though the phenomena of stochastic resonance has been seen in biological experiments and in slow environmental changes, engineering applications based on this has been a few^9–15^. The fundamental principle of harvesting the noise power to empower the signal or the information content of the input can prove quite useful if used for practical purposes. One use of this, which is quite evident from our experiments is that it can be used to detect weak signals in presence of noise. Another interesting application that we discuss below in detail is to preserve the frequency spectra of the input signal after thresholding as shown in^23^. This finds application in cochlear implants where high-fidelity can be maintained without amplifying the signal, that can cause harm to the patient. The frequency spectra for each vowel sound has a special nature. It has a fundamental frequency along with its harmonics, but most of the power is concentrated at one particular frequency often called ‘formant’ as shown in the Fig. 6B below. When a vowel is transmitted through the auditory nerve it generates an action potential for the nerve if its amplitude is more than a certain value. If we want to replicate the action of auditory nerves with the help of a cochlear implant the shape of the spectrum is not preserved after the thresholding as it is a non-linear process, and we get a signal with power mostly at the fundamental frequency (Fig. 6E).

This can be overcome in two ways, either by increasing the signal power, or by adding noise illustrated in Fig. 6. Increasing signal power by too much can cause pain in the ear and impair hearing, therefore not deemed suitable for our purposes. But by adding some small amount of noise along with the signal we make the system stochastic rather than deterministic. The probability of crossing the threshold then becomes dependent on the power that a particular frequency has in the input and hence the output reflects the input pattern as shown in the Fig. 6G,J,L. Similar to our previous discussions the amount of noise needed to best preserve the original structure is neither too low so as to not change the structure at all, or too high so that only the noise dictates the output. These two extreme cases along with the case where a moderate amount of noise is able to reconstruct the original signal is shown in Fig. 6F,H,G respectively. The amplitude of the output spectrum at the formant frequency vs the noise power is also shown in Fig. 6C,D for two different peak-to-peak input vowel amplitudes (100 and 50 mV respectively). Here the original vowel signal (Fig. 6A,B) is presented at the input of our circuit after appropriate amplification along with a bias ${V}_{dc}$. The amount of noise needed to preserve the signal structure depends on the amplitude of the signal, as the signal power goes up so does the amount of noise needed to get the similar output. Other two vowels are also shown in the subsequent figures, and also the output when the noise is similar to that used to extract the vowel ‘ae’, this means the same level of noise can be used to extract the vowel sounds without any prior knowledge of what sound it is.

**Figure 7:**
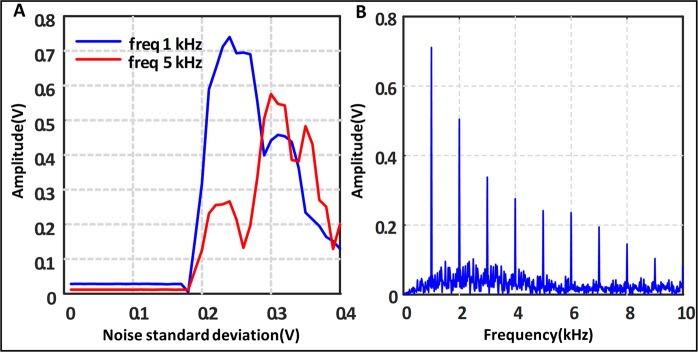
Output with two frequencies at the output. The input is sum of two frequencies 1 and 5 kHz with equal 10 mV amplitude. (**A**) Shows the output at those frequencies for different noises. (**B**) Shows the output spectra with noise fixed at 0.25.

Another potential application that has been studied utilizes the nature of frequency response that we see for the circuit. We see that the device in this setup acts as a non-linear low-pass filter. The low-pass nature is attributed from two facts; one as frequency increases the gain of the system decreases and the same amount of noise is insufficient for resonance. Second, as the frequency increases the number of spikes per period decreases dramatically as the time period becomes comparable to the charging-discharging time of the capacitor itself. Also the frequency range that it amplifies can be determined by the amount of noise applied to its input. Hence, by changing just the amount of noise, particular high frequencies can be rejected as we experimentally note. Traditionally, changing the cutoff frequency of an analog filter requires a hardware change, but here we note that by changing the noise power we can shift the cutoff frequency. As a demonstration, we apply two sinusoids with frequencies 1 and 5 kHz to the system. We observe that the 5 kHz frequency is suppressed at the output showing that the circuit can be used to reject high frequency noise by adjusting the noise power. We can see from Fig. 5 that the range of frequencies that gets amplified by the SR action depends on the noise level as well as the fundamental frequency of the system which is governed by the internal capacitance of the IMT device. By choosing the internal capacitance appropriately we can have a low pass filter whose cut-off frequency can be changed by the amount of noise added to the system up to a certain extent (Fig. 7).

## Materials and Methods

### Samples

Vanadium dioxide (VO_2_) is grown to a 10 nm thickness on a substrate of (001) TiO_2_ with molecular beam epitaxy^24^. The devices widths are defined by dry etching with CF_4_ and device lengths by the Pd/Au metal contacts which are deposited using electron beam evaporation. The VO_2_ devices varied in length from 100 nm to 1um with resulting insulator-to-metal transition threshold voltages ranging from 0.7 V to 6 V. The largest devices of size 1 × 2 *μm*^2^ with relaxation oscillations of ~5 V amplitude were used to perform the experiments in order to maximize the small-signal amplification.

### Experimental setup

A single IMT oscillator is realized by connecting the two-terminal device in series with an external n-channel MOSFET. A V_DD_ of 7 V and a gate voltage of 0.81 V was used to bias the transistor such that the load line passed through only the insulating region (Fig. 2B). An additional 200 mV of gate voltage would cause continuous relaxation oscillations. All experiments were performed by adding noise to the gate signal of the series transistor. The small-signal sinusoidal wave and noise were generated and combined using the Keysight 81150 A Function Generator. The intrinsic ${v}_{h}$ noise standard deviation of the oscillator was measured to be 85 mV which is comparable to the standard deviation of the added gate noise and needs to be accounted for when biasing the transistor. The vowel sounds used in this paper were obtained from the North Texas vowel database^25^. Each vowel waveform was normalized to a 100 mV peak-to-peak signal on top of the gate voltage bias along with any added noise.

### Simulations

The modelling and simulations were done in MATLAB. The device characteristics and the noise values were measured and the data analyzed, to approximate the different device variables and noise magnitudes. The MOSFET was assumed to be in saturation throughout the operation and the IMT itself were modelled with hysteresis with different behavior in the insulating and metallic regions.

## Conclusion

In this article, we explore stochastic resonance (SR) in a simple electronic circuit and demonstrate particular applications of SR. For vowel sound enhancement, we have seen how adding noise to the system helps preserve the primary formant power and the relative structure of the sound after it passes through the non-linearity inherent to the system. The circuit imitates a neuron carrying a signal and we need not increases the signal power at all but add a little amount of noise to pass through the synaptic/thresholding nature of the system and still preserve the nature of the sound.

It is evident from the output amplitudes vs frequency plots that SR exhibits a form of low pass filtering, and the cut-off frequency for the low-pass filter is determined by not only the circuit parameters (namely the capacitance and resistance) but also by the amount of noise added to the system. By changing the noise power, we can change the cut-off frequency and use this to our advantage to reject different ranges of frequency without requiring any hardware changes.

In summary, we present a simple electronic implementation of SR and demonstrate experimentally and theoretically how system noise can be harnessed to provide exciting opportunities for analog signal processing.
